# Mixed intestinal protozoal infection masquerading as ulcerative colitis in advanced AIDS: a case report

**DOI:** 10.3389/fmed.2026.1895591

**Published:** 2026-07-01

**Authors:** Yan Zhang, Xiaojin Ding, Jing Gao, Kenv Pan, Aifang Xu

**Affiliations:** 1 Department of Clinical Laboratory, Hangzhou Xixi Hospital, Hangzhou Sixth People's Hospital, Hangzhou, Zhejiang, China; 2 Department II of Infectious Diseases, Hangzhou Xixi Hospital, Hangzhou Sixth People's Hospital, Hangzhou, Zhejiang, China; 3 Department of Pathology, Hangzhou Xixi Hospital, Hangzhou Sixth People's Hospital, Hangzhou, Zhejiang, China

**Keywords:** AIDS, case report, *Entamoeba histolytica*, *Giardia lamblia*, ulcerative colitis

## Abstract

Co-infection with *Entamoeba histolytica* (*E. histolytica*) and *Giardia lamblia* (*G. lamblia*) is exceptionally rare reported in individuals with human immunodeficiency virus (HIV), despite both protozoa being well-recognized etiologic agents of intestinal disease in the general population. The overlapping clinical manifestations of protozoal infections and inflammatory bowel disease may pose significant diagnostic challenges, particularly in patients with advanced immunosuppression. We report the case of a 34-year-old man with advanced acquired immunodeficiency syndrome (AIDS) who presented with persistent mucopurulent bloody stools for 2 months. Upon evaluation in our gastroenterology department, ulcerative colitis was initially considered; however, stool microscopy subsequently identified trophozoites of *E. histolytica* and *G. lamblia*. Colonoscopic assessment, supported by histopathological examination of biopsy specimens, confirmed *E. histolytica* infection. The patient was initiated on intravenous metronidazole in conjunction with antiretroviral therapy, leading to a gradual improvement of symptoms, with resolution of diarrhea within 10 days. This case highlights the importance of considering mixed protozoal infections in the differential diagnosis of chronic dysentery among immunocompromised patients. It further underscores the critical diagnostic value of repeated stool microscopy and histopathologic confirmation to avoid misdiagnosis and prevent delays in appropriate therapy.

## Introduction

*Entamoeba histolytica* (*E. histolytica*) is a highly pathogenic intestinal protozoan responsible for amoebic dysentery, amoebic liver abscess, and other serious diseases, ranking as the second leading cause of parasitic infection-related deaths worldwide. In contrast, *Giardia lamblia* (*G. lamblia*) typically causes chronic diarrhea and malabsorption syndrome (1–3). Reports focusing on concurrent *E. histolytica* and *G. lamblia* infection remain limited, particularly in patients with advanced acquired immunodeficiency syndrome (AIDS). The co-occurrence of HIV and multiple parasitic infections not only exacerbates the clinical severity of protozoan infections but also accelerates HIV progression to AIDS (4–8). In regions with low HIV prevalence, the complications of HIV/AIDS complicated by intestinal protozoan infections often receive insufficient attention, leading to underestimations of their potential harm. The clinical symptoms of amoebic dysentery and ulcerative colitis overlap significantly, as both conditions present with abdominal pain, diarrhea, mucus, and bloody stools, increasing the risk of misdiagnosis. Intestinal parasitic infections remain an important cause of morbidity among people living with HIV, particularly in individuals with advanced immunosuppression and in resource-limited settings. Such infections may contribute to chronic diarrhea, malnutrition, impaired quality of life, and adverse clinical outcomes (9). Therefore, timely and accurate diagnosis, along with standardized treatment of intestinal protozoan infections in HIV patients, is vital for improving patient prognosis.

## Case presentation

A 34-year-old office worker presented to the gastroenterology department with a two-month history of persistent mucopurulent bloody stools, occurring 5–7 times daily. These symptoms, of unknown etiology, were accompanied by abdominal distension, lower abdominal pain, tenesmus, purulent sputum production, bilateral lower limb weakness, and significant weight loss (approximately 13 kg during the preceding 2 months). He was diagnosed with HIV infection 1 month before and had not previously received antiretroviral therapy. At a local hospital, he received treatment with montmorillonite powder for diarrhea and live *Bacillus licheniformis* capsules to regulate gut flora, but with minimal efficacy.

**Figure 1:**
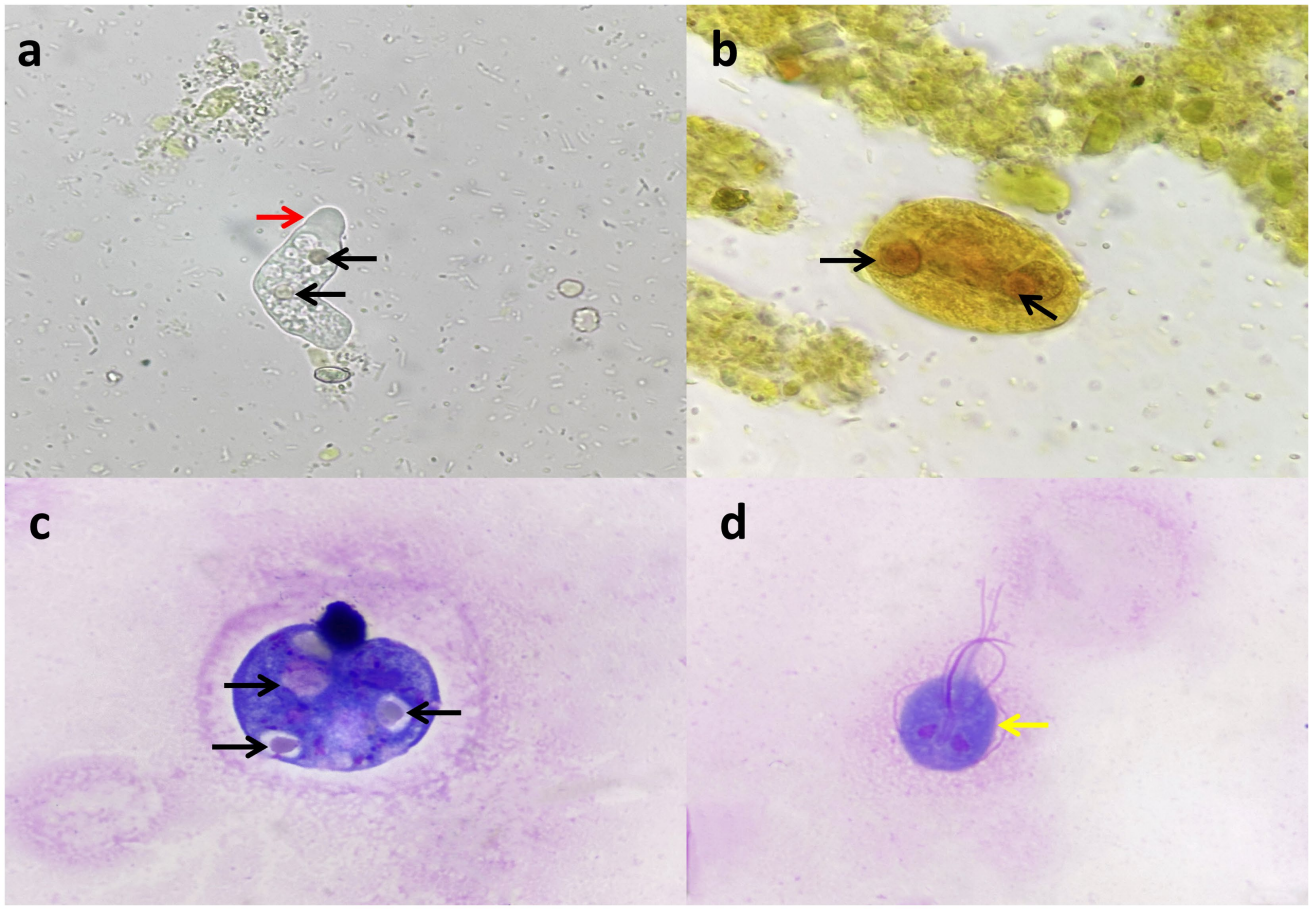
**(a)** *E. histolytica* trophozoite extending pseudopodia (red arrow) and phagocytizing red blood cells (black arrow) (saline smear, × 400). **(b)** *E. histolytica* trophozoite phagocytizing red blood cells (black arrow) (Iodine stain, × 1,000). **(c)** *E. histolytica* trophozoite phagocytizing red blood cells (black arrow) (Wright Giemsa stain, × 1,000). **(d)** *G. lamblia* trophozoite showing a pear-shaped body, two symmetrical nuclei, four pairs of flagella, and a ventral adhesive disc (yellow arrow) (Wright Giemsa stain, × 1,000).

**Figure 2:**
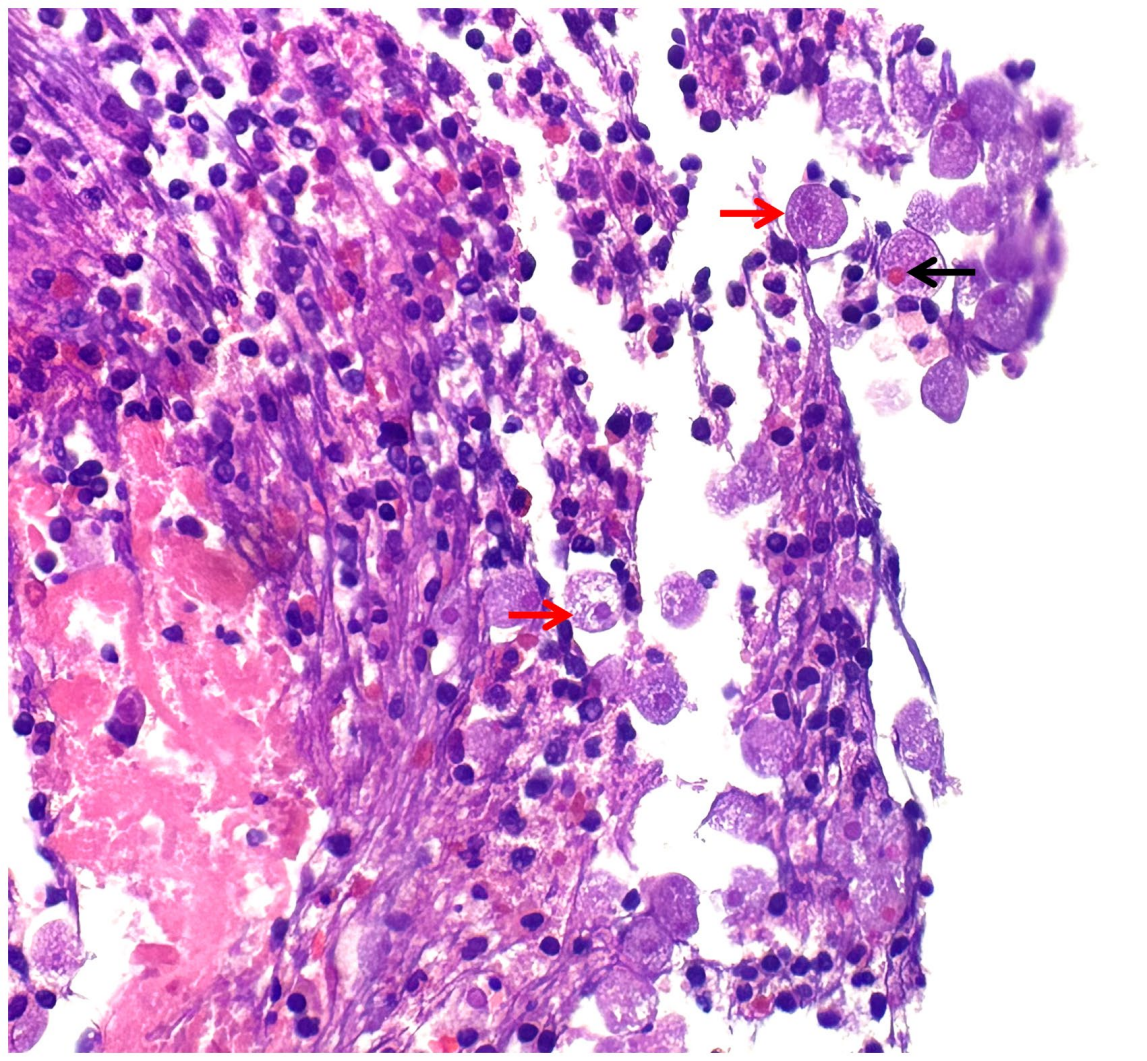
Amoeba trophozoites are round or oval, with single, small and round nuclei (red arrow) and PAS positive cytoplasm, and phagocytic erythrocytes can be seen (black arrow) (periodic acid-Schiff stain, × 400).

The patient’s clinical examination results from the outside hospital, together with those obtained after the current admission, are summarized in Table 1. The patient presented with mild leukopenia (3.00 × 10^9/L), hypoalbuminemia (35.8 g/L), and strongly positive fecal occult blood testing (+++). HIV serology was positive. During hospitalization, inflammatory markers, including CRP (21.87 mg/L) and ESR (23 mm/h), were elevated, whereas fecal occult blood remained positive, consistent with ongoing intestinal mucosal injury. Upon admission, the patient was initially diagnosed with ulcerative colitis. Two stool specimens collected on August 13 and August 18, 2025, showed positive fecal occult blood test results of (++) and (+), respectively, without evidence of intestinal parasites. Each freshly collected specimen underwent immediate direct saline wet-mount microscopy. On August 19, 2025, motile trophozoites morphologically consistent with *E. histolytica* were identified during stool examination, prompting repeat specimen collection for confirmation. Both samples were yellow, loose stools and tested positive for fecal occult blood (++). Microscopic examination revealed 0–4 WBCs and 32–62 RBCs per HPF. The saline smear tests unexpectedly revealed a motile trophozoite of *E. histolytica,* extending pseudopodia and phagocytizing two RBCs (Figure 1a). Iodine staining confirmed a pale brown *E. histolytica* trophozoite and swallowed two RBCs (Figure 1b). Wright–Giemsa staining demonstrated an *E. histolytica* trophozoite containing multiple ingested erythrocytes (Figure 1c) and a typical *G. lamblia* trophozoite exhibiting a pear-shaped morphology, bilateral symmetry, and two prominent nuclei (Figure 1d). Colonoscopy with biopsy indicated chronic active inflammation with erosions in the transverse colon and chronic inflammation in the descending colon. Inflammatory necrotic material revealed periodic acid-Schiff (PAS)-positive structures, amoeba trophozoites are round or oval, with single, small and round nuclei and phagocytic erythrocytes can be seen, consistent with *E. histolytica* trophozoites (Figure 2).

**Table 1:** Comparison of laboratory findings between the referring hospital and the current admission.

Variable	Outside hospital (2025-07-01)	Current admission (2025-08-13)	Reference range
WBC count (×10^9/^L)	3.00	4.58	(3.50–9.50)
Hemoglobin (g/L)	139	148	(130–175)
Eosinophil count (×10^9^/L)	0.15	0.18	(0.02–0.52)
PLT count (×10^9^/L)	203	300	(125–350)
PT (S)	Normal	11.4	(9.7–12.6)
APTT (S)	Normal	28.3	(23–32.6)
TT (S)	Normal	17.4	(14–21.0)
Fibrinogen (g/L)	Normal	4.23	(1.8–3.5)
D-D dimer (mg/L FEU)	Normal	0.73	(0.00–0.55)
AST (U/L)	Normal	19	(15–40)
ALT (U/L)	Normal	11	(9–50)
Creatinine (μmol/L)	/	87	(57–111)
BUN (mmol/L)	/	4.2	(3.1–8.0)
Albumin (g/L)	35.8	38.0	(40–55)
CRP (mg/L)	3.00	21.87	(0–10)
ESR (mm/h)	/	23	(0–15)
HIV Ab	Positive	Positive	Negative
FOB	+++	++	Negative

WBC, white blood cell; PT, prothrombin time; APTT, activated partial prothromboplastin time; TT, thrombin time; AST, aspartate aminotransferase; ALT, alanine aminotransferase; BUN, blood urea nitrogen; CRP, C-reactive protein; ESR, erythrocyte sedimentation rate; HIV-Ab, human immunodeficiency virus antibody; FOB, fecal occult blood.

**Figure 3:**
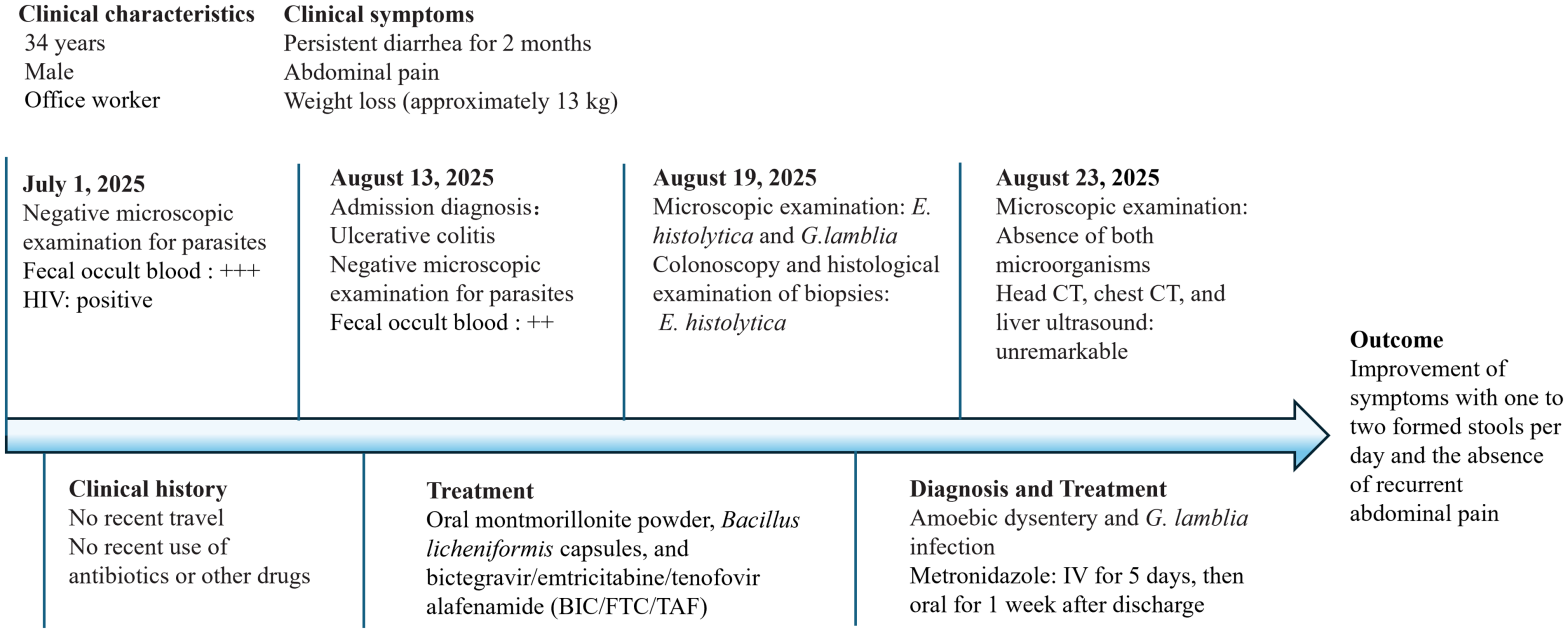
Timeline of the patient’s clinical history.

Further evaluation revealed advanced HIV infection, characterized by a high HIV RNA level (3.49 × 10^9^ copies/L) and a markedly reduced CD4^+^ T-lymphocyte count of 32 cells/μL. The revised diagnosis included AIDS, amoebic dysentery, and *G. lamblia* infection. The patient was treated with intravenous metronidazole (0.5 g every 8 h) for anti-amoebic therapy, as well as ongoing antiretroviral therapy (ART). After 3 days, a repeat stool examination showed a positive occult blood test (2+), but no parasites were detected. By day ten, the patient’s diarrhea had resolved, the fecal occult blood test returned negative, and no parasites were found, leading to significant symptom improvement and eventual discharge. Oral metronidazole was continued for 1 week after discharge. The complete eradication of the two pathogens was linked to an improvement in their symptoms. The patient reported one to two formed stools per day and an absence of recurrent abdominal pain. Figure 3 summarizes the patient’s full clinical history.

## Discussion

Mixed intestinal protozoal infections involving *E. histolytica* and *G. lamblia* have rarely been reported, particularly in patients with advanced HIV infection. In the present case, microscopic examination, together with colonoscopic and histopathological findings, established the diagnosis of concurrent amoebiasis and giardiasis. Although both parasites are common causes of intestinal protozoal disease worldwide, reports of concomitant infection remain limited, and the true prevalence may be underestimated because routine diagnostic investigations often focus on a single pathogen.

Diarrhea is one of the most frequent gastrointestinal symptoms in HIV-infected individuals, occurring in up to 90% of cases (1). HIV predisposes individuals to opportunistic infections through progressive impairment of cellular immunity (2). Although the prevalence of opportunistic gastrointestinal diseases has decreased with the introduction of ART, HIV/AIDS patients still experience an elevated risk of parasitic infections due to ongoing immune system damage (10).

*E. histolytica* is a protozoan parasite and remains the only intestinal amoeba definitively proven to cause invasive disease in humans. It is primarily spread through contaminated food and water, particularly in areas with poor sanitation, such as South America, Africa, and Asia (11, 12). In HIV-positive individuals, the infection frequently progresses to invasive amoebiasis, often manifesting as amoebic colitis or liver abscess. This progression may be linked to the interaction between amoeba surface lectins and HIV replication, exacerbating immune damage and hastening AIDS progression (5). The incidence of amoebiasis has notably risen among men who have sex with men; without prompt diagnosis and treatment, invasive amoebiasis related to HIV can lead to severe complications and heightened mortality rates (13–15).

*G. lamblia* is an important enteric protozoan pathogen that may cause more severe or prolonged disease in immunocompromised individuals. As the third most common cause of diarrhea globally, *G. lamblia* infections are significantly more prevalent in developing countries compared to developed nations (3, 11, 16). Co-infections of *E. histolytica* and *G. lamblia* are exceedingly rare in clinical practice, and this case highlights how HIV-induced immune deficiency may predispose patients to such mixed protozoal infections. Research indicates a low CD4^+^ T cell count is a critical risk factor for opportunistic infections; specifically, a CD4^+^ T cell count below 200 cells/μL markedly increases the risk of *E. histolytica* infections (8, 12). The patient’s low CD4^+^ T cell count of 32 cells/μL indicated severe immune deficiency, contributing to the mixed protozoal infection.

In immunocompromised patients, amebic infections often present atypically, complicating differentiating diagnoses. Misdiagnosing an amebic infection as ulcerative colitis can lead to treatment delays and inappropriate corticosteroid therapy, worsening the infection. Stool microscopy remains a practical, inexpensive, and widely used diagnostic method for intestinal protozoal infections, particularly in resource-limited settings. However, its diagnostic performance is limited by variable sensitivity and specificity, and species-level identification may require antigen detection assays, molecular testing, or histopathological examination. In the present case, both *E. histolytica* and *G. lamblia* were identified only after repeated stool examinations. Ultimately, the diagnosis was supported not only by stool microscopic findings but also by the identification of PAS-positive trophozoite-like organisms in colonic biopsy specimens, together with compatible clinical manifestations and a favorable response to metronidazole therapy. In clinical practice, it is advisable for patients suspected of having inflammatory bowel disease to collect fecal samples across consecutive days to enhance detection rates. Notably, eosinophilia was absent in this patient, consistent with previous observations that eosinophil responses are often limited in protozoal infections and may be further blunted in advanced HIV infection.

Previous studies have reported intestinal protozoal infections in people living with HIV, including isolated *E. histolytica* or *G. lamblia* infection, as well as occasional cases of multiple concurrent parasitic infections. A recent case report described an HIV-infected patient with simultaneous infection by *E. histolytica*, *G. lamblia*, *Cryptosporidium* species, and *Cystoisospora* species (17). However, reports focusing on concurrent *E. histolytica* and *G. lamblia* infection in patients with advanced HIV disease remain limited. This patient presented with severe immunosuppression, endoscopic findings mimicking inflammatory bowel disease, and histopathological evidence of invasive amoebiasis, highlighting the diagnostic challenges posed by mixed intestinal protozoal infections in advanced HIV infection.

A limitation of this case is the absence of molecular confirmation by PCR or antigen detection assays for Entamoeba and Giardia species. Although PAS-positive trophozoite-like organisms were identified in colonic biopsy specimens, suggestive of invasive amoebiasis, microscopy and histopathology alone cannot definitively distinguish *E. histolytica* from morphologically similar non-pathogenic species such as *E. dispar* and *E. moshkovskii*. In addition, stool microscopic examination revealed trophozoites of *G. lamblia* exhibiting characteristic “face-like” morphology, further supporting the presence of intestinal protozoal infection; however, species-level confirmation was not performed using molecular methods. Nevertheless, the histopathological findings, together with the clinical presentation and favorable response to metronidazole-based therapy, strongly supported the diagnosis of amoebic dysentery and *G. lamblia* infection.

Metronidazole remains a first-line treatment for both invasive amoebiasis and giardiasis. In the present case, the patient received intravenous metronidazole for 5 days followed by oral metronidazole for 7 days, with marked clinical improvement and no evidence of recurrence was observed during follow-up. However, a luminal agent was not administered, which should be acknowledged as a limitation. Notably, the metronidazole regimen was also expected to provide adequate treatment for the concurrent *G. lamblia* infection.

## Conclusion

This case highlights that mixed *E. histolytica and G. lamblia* infection can mimic ulcerative colitis in patients with advanced HIV infection. In HIV-infected individuals presenting with chronic bloody diarrhea, parasitic infections should be carefully considered in the differential diagnosis, even when endoscopic findings suggest inflammatory bowel disease. Early parasitological evaluation is essential for accurate diagnosis and timely targeted treatment.

## Patient perspective

Patient was discharged in stable condition after 10 days. The patient was satisfied with our management as his clinical symptoms resolved with therapy.

## Data Availability

The original contributions presented in the study are included in the article/supplementary material, further inquiries can be directed to the corresponding author.
